# Noncovalent Functionalization of Graphene in Suspension

**DOI:** 10.1155/2013/656185

**Published:** 2013-03-28

**Authors:** Wenzhi Yang, Sultan Akhtar, Klaus Leifer, Helena Grennberg

**Affiliations:** ^1^Department of Chemistry-BMC, Uppsala University, P.O. Box 576, 751 23 Uppsala, Sweden; ^2^Department of Engineering Sciences, Electron Microscopy and Nanoengineering, Uppsala University, P.O. Box 534, 751 21 Uppsala, Sweden

## Abstract

Suspensions of graphene, prepared from graphite foil by sonochemical exfoliation, have been treated with new nonpolar pyrenebutyric amides. The assemblies, in suspension and after deposition on solid supports, were characterized by NMR, absorption, and fluorescence spectroscopy and by transmission electron microscopy, where the well-defined shape and size of an appended [60]fulleropyrrolidine unit facilitates TEM detection of the nonstationary molecules. The accumulated evidence, also including direct comparisons of carbon nanotubes treated with pyrene amides under the same conditions, proves the successful noncovalent functionalization of graphene suspended in non-polar solvent with non-polar pyrene derivatives.

## 1. Introduction

Graphene, the two-dimensional parent structural unit of three-dimensional graphite and one-dimensional carbon nanotubes [1, 2], has been treated theoretically since decades, with suggestions of numerous applications that would benefit from the predicted unusual electron transport properties of a defect-free extended delocalized aromatic carbon system [3]. When the material was shown to exist, a new expansive area opened, involving also experimentalists since reproducible production of high-quality graphene and controlled modification thereof are keys to any of the suggested applications [4]. The strategies include molecular synthesis [5] but the main ones are still micromechanical exfoliation from HOPG, [1–3] epitaxial growth on SiC surfaces [6], chemical vapour deposition on metals [7, 8], and chemical exfoliation, either via graphitic and graphene oxides [9, 10] or in direct sonochemical processes not involving any oxidative, acidic or reductive reagents [11, 12]. The latter gives, in a controllable and scalable fashion, dispersions/suspensions of graphene flakes well suited for further chemical manipulation, where protocols developed for carbon nanotube functionalizations have been obvious starting points [13–15]. Since covalent functionalization of the largely planar unsaturated carbon system introduces sp^3^ sites and by this permanent change of the electronic properties, such routes would be interesting mainly for permanent doping purposes [16, 17]. In contrast, non covalent routes would inflict only minor and temporary changes to the graphene *π*-system and, as has been demonstrated for carbon nanotubes [18, 19], render possible the introduction of almost any functional unit in a potentially reversible fashion. Such strategies have been reported for graphene oxide [20, 21], for solid films of reduced graphene oxide [22] as well as for micromechanically cleaved graphene deposited on SiO_2_ [23, 24], and for graphene grown epitaxially on Ru [25]. An additional advantage of the noncovalent strategy is the predicted possibility to fine-tune the transport properties [26]. For graphene *suspensions*, interactions that result in transfer of the graphene from a nonpolar to a polar phase are unambiguous as the result is observable without any instrument [21, 27], whereas interactions between graphene and noncharged nonpolar molecules in nonpolar media to a higher extent rely on indirect evidence [28, 29]. In the present paper, we have used nonpolar pyrenes designed primarily for proving the interaction and not for giving “supramolecular” properties to the graphene. The evidence from transmission electron microscopy (TEM), supported by NMR and UV-Vis fluorescence spectroscopy, proves that graphene suspended in a nonpolar aromatic solvent can be noncovalently functionalized by neutral pyrene derivatives.

## 2. Results and Discussion

**Figure 1:**
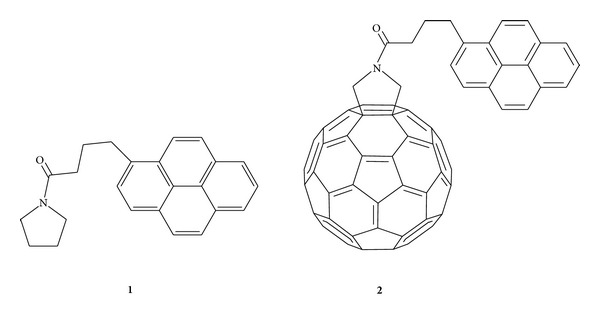
Pyrene derivatives **1** and **2** prepared and used for functionalization of graphene suspensions in this study.

Two pyrene derivatives were prepared for the study (Figure 1). The pyrrolidine derivative **1** was prepared in good yield from 4-pyrenebutyric acid chloride [30] and pyrrolidine. The corresponding [60]fulleropyrrolidine compound **2** was obtained in moderate yield from [60]fullerene and 4-pyrenebutyric acid chloride in a two-step-one-pot procedure. The purified products were fully characterized by IR, NMR, UV-Vis absorption, and fluorescence spectroscopy.

**Figure 2:**
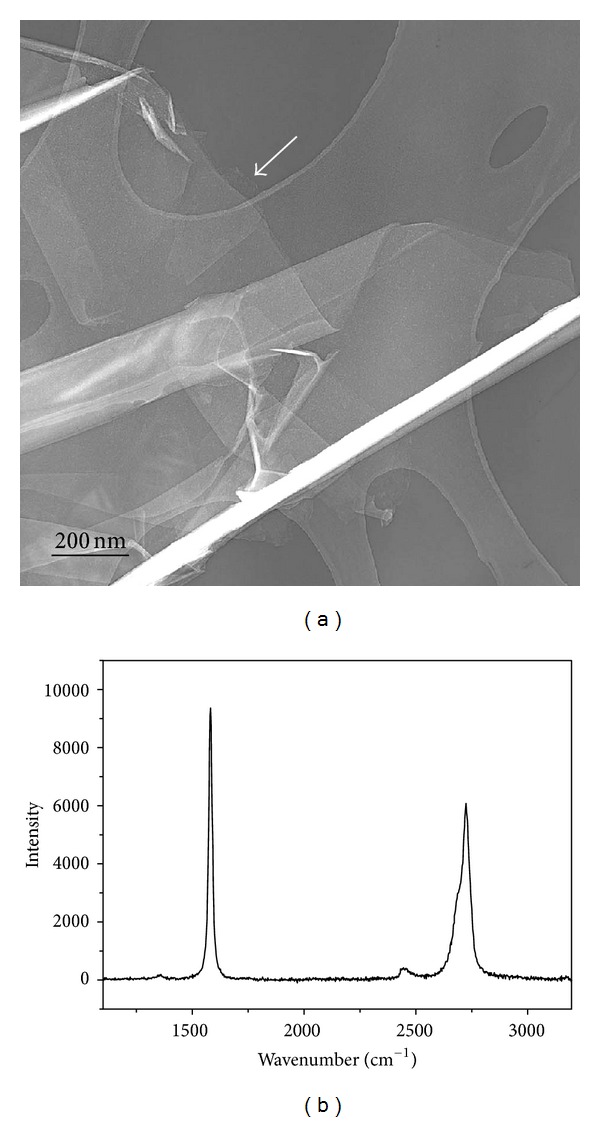
(a) Overview TEM of as-prepared graphene dip-deposited from toluene, the arrow pointing to a flake edge suspended over a hole in the grid, a suitable point of analysis by HR-TEM, and (b) Raman spectrum (514 nm) of drop-deposited graphene flakes from the same preparation. Both analyses prove the presence of few-layer graphene.

Graphene suspensions were obtained from graphite foil by sonication in an organic solvent. Overview TEM (Figure 2(a)) of dip-deposited as-prepared graphene showed folded flakes with an average thickness of 5-6 nm, with less than 3–5 layers at the nonfolded borders. The thinner flakes were more extensively folded. Raman analysis of drop-deposited samples displayed the expected G and 2D features, indicating high-quality few-layer graphene (Figure 2(b)). The suspensions of graphene prepared in toluene, isopropanol, and chloroform are stable over many hours, which allows for spectroscopic studies of the functionalization process.

**Figure 3:**
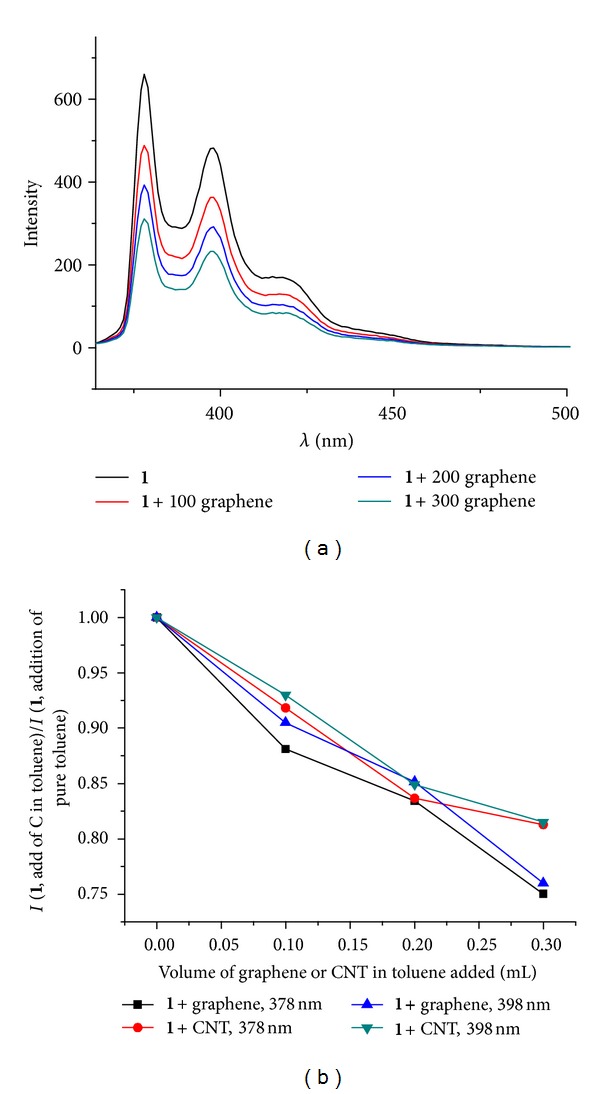
(a) Fluorescence titration (*λ*_exc_ = 344 nm) adding 100/200/300 *μ*L of a 0.2 mg/mL graphene suspension in toluene to a solution of **1** in toluene, [1]_initial_ ≈ 6.26 × 10^−6^ mol/L. (b) Normalized titration data at two fluorescence wavelengths. Intensity of (**1** + volume of graphene or MWCNT suspension)/Intensity of (**1 **+ volume of solvent).

Fluorescence titrations monitoring the interaction between graphene and **1** were carried out for toluene and isopropanol suspensions of graphene. None of the graphene suspensions exhibited fluorescence in the 350–450 nm area where the fluorescence from **1** is most prominent. The presence of **1** was evident in all experiments, which is in contrast to the near-complete quenching of pyrene fluorescence that has been observed for in particular single-wall carbon nanotube systems [18, 19]. Still, the fluorescence intensity of **1** decreases more on addition of graphene suspension than when adding pure solvent which is a strong indication that the pyrene and the graphene are interacting (Figure 3). Very similar trends, although not exhibiting saturation, were observed also in control experiments using suspensions with comparable mass loading of nonfunctionalized purified large-diameter MWCNTs as well as in studies using isopropanolic suspensions/solutions where no competing *π*-*π* interactions from the solvent are present [28].

**Figure 4:**
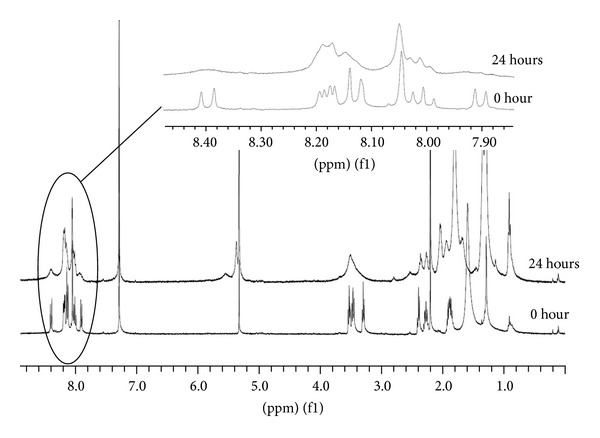
^1^H NMR of **1** in CDCl_3_ before (lower trace) and 24 h after addition of graphene (upper trace).

Graphene suspensions in chloroform exhibited a higher degree of scattering, and the reproducibility of the fluorescence titration experiments was hence not satisfactory. In contrast, analysis by NMR spectroscopy was feasible. The ^1^H NMR spectrum of **1** treated with graphene displays broader aromatic signals than the spectrum of pure **1** (Figure 4) which is significant for noncovalently attached pyrene in exchange with the substantial excess of free pyrene [18, 19]. 

The limited solubility and efficient intramolecular fluorescence quenching of **2** make spectroscopic detection of functionalization less straight-forward than those for the more readily available **1**. However, the [60]fullerene unit of **2** should be possible to identify by TEM, a method that would provide direct proof of functionalization additional to that obtained from the spectroscopic studies. For the TEM analyses, graphene in chloroform or toluene was treated with **2**. Prior to dip-deposition of the graphene onto standard carbon-coated TEM grids, the reactions were subjected to a workup protocol involving filtrations in order to remove excess **2** and resuspension of functionalized graphene in fresh solvent. Controls consisting of graphene treated with pure solvent or with [60]fullerene solutions were prepared using the same protocol. The yield of functionalization was higher in both solvents using the [60]fullerene-pyrene derivative **2** than using [60]fullerene, as determined by UV-Vis spectroscopy of the solvents removed in the filtration step.

**Figure 5:**
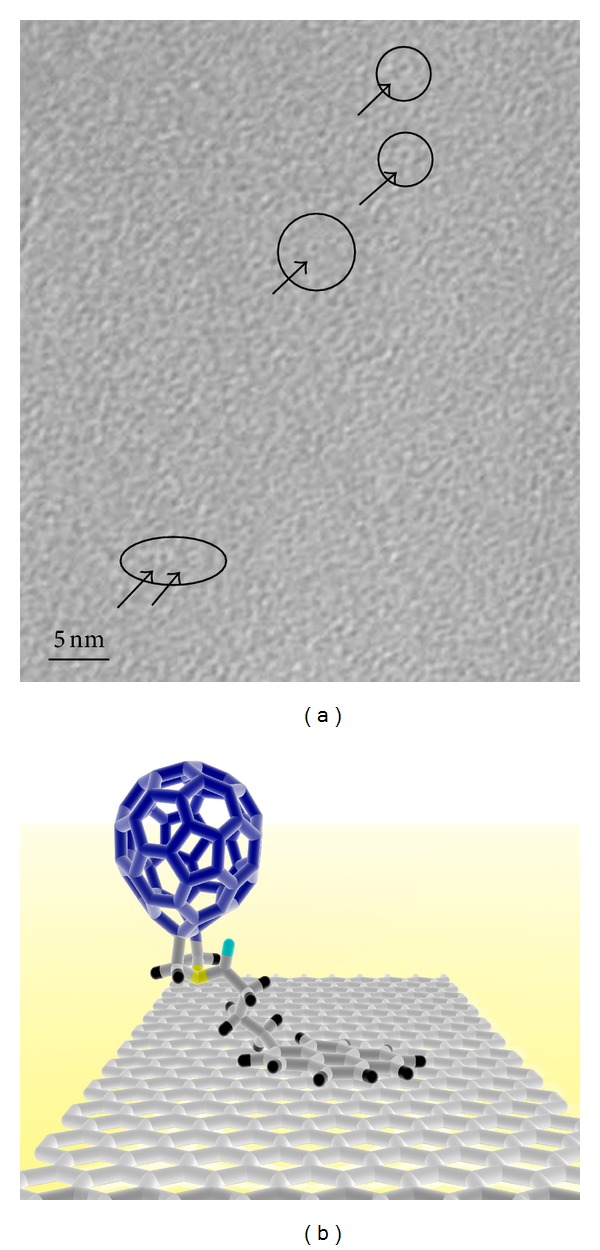
(a) HR-TEM of graphene treated with **2 **in toluene. Scale bar corresponds to 5 nm. Arrows and black ovals indicates some of the large number of [60]fullerene candidates distributed over the surface, all of which appear brighter than the background. The contrast was enhanced digitally. (b) Cartoon depicting **2** on a graphene surface, consistent with the observations of the [60]fullerene-graphene and [60]fullerene-pyrene-graphene systems.

High-resolution TEM (Figure 5) of nonfolded regions of the graphene flakes that span over holes in the carbon support grid and including digital enhancement of the images revealed that the surface of the graphene treated with **2** displayed circular structures with a diameter of ca. 1 nm, that is, fullerene candidates [31, 32]. The surface of the control was much more crystalline, and the circular structures were not seen. The samples treated with nonfunctionalized [60]fullerene have a surface morphology similar to that of the nontreated control sample, that is, island structures and negligible amorphous coverage on the flakes, except for some circular structures at the more amorphous edge areas of the flakes. This is consistent with the UV-Vis analysis of the filtrates obtained after removal of the functionalized graphene and supporting the assumption that binding of **2 **is primarily a result of interaction between graphene and the pyrene (16 carbon atoms) and not to the considerably smaller available contact area of the [60]fullerene (6 carbon atoms) [33]. 

Due to the dynamic nature of the noncovalent assemblies and the complexity of the image acquisition in order to minimize damage by the electron beam, a more quantitative evaluation also involving TEM contrast simulations has not yet been carried out. 

## 3. Conclusion

We have obtained direct proof of noncovalent functionalization of graphene in organic suspension employing two new nonpolar pyrenes as functionalization agents and graphene material produced from high-quality graphite foil by a facile sonochemical exfoliation. The success of the solvent-phase graphene functionalization has been proven by NMR, absorption, and fluorescence spectroscopy using a pyrenebutyric amide and, to the best of our knowledge, for the first time by transmission electron microscopy using a pyrene with an appended [60]fulleropyrrolidine unit. The results are fundamentally important for future scaleable developments of functional supramolecular materials based on high-quality graphene and the numerous libraries of nonpolar pyrene derivatives.

## 4. Experimental

### 4.1. General Details

Reagents were from commercial sources and were used without further purification unless stated otherwise. Graphite foil (Alfa Aesar, 99.8%, metal basis, thickness of 0.5 mm) was used for the preparation of graphene. Multiwalled carbon nanotubes (MWCNTs), diameter 60–100 nm, were from NTP (Shenzhen Nanotech Port Co., Ltd.). The MWCNTs were purified using a reagent-free microwave-assisted protocol [34] prior to the noncovalent functionalization, and 4-pyrene-1-yl-butyroyl chloride was prepared according to a literature procedure [30]. Toluene used in reactions was dried over molecular sieves for 3 days. DCM used in reactions was freshly distilled from CaH_2_. Acid was removed from CHCl_3_ by filtration through basic Al_2_O_3_ immediately before use. CDCl_3_, stabilized by Ag foil, was used as received. Column chromatography was performed using Matrix Silica 60A/35-70 micron as solid phase. Thin-layer chromatography (TLC) was performed on Merck precoated silica gel 60-F254 plates. 

^1^H NMR and ^13^C NMR spectra were recorded on a Varian Unity 400 spectrometer. The chemical shifts are reported using the residual solvent signal as an indirect reference to TMS: CDCl_3_ 7.26 ppm (^1^H) and 77.0 ppm (^13^C). Raman spectra were recorded on a Renishaw Raman spectrometer using a 514 nm Argon laser and a 50x lens. Fluorescence spectra (excitation at 344 nm) were recorded on a Varian Cary Eclipse fluorescence spectrometer and UV spectra were recorded on a Varian Cary 3 Bio UV-Vis spectrometer. Transmission electron microscopy was performed on a JEOL 2000FXII operating at 200 kV.

### 4.2. Synthesis of 4-(pyrene-1-yl)-1-(pyrrolidine-1-yl)butan-1-one (**1**) 

Pyrrolidine (47 mg, 0.66 mmol) was dissolved in dry DCM (2 mL) under N_2_ atmosphere. The solution was cooled to 0°C and pyridine (0.45mL, 4.5 mmol) was added dropwise followed by addition of 4-(pyren-1-yl)butanoyl chloride (63 mg, 0.06 mmol) in DCM (3 mL). The ice bath was removed and the reaction was stirred for 17 hours at room temperature. The mixture was then washed with aq. sat NaHCO_3_. The organic phase was dried over MgSO_4_ and the solvent was remover in vacuo. The residue was purified by column chromatography (DCM to DCM/ethyl acetate = 20 : 1). The product was obtained as a yellow solid (23.0 mg, 32%). ^1^H NMR (400 MHz, CDCl_3_), *δ* (ppm): 8.39 (pyrene C–H, d, 1H, *J* = 9.2 Hz), 8.19 (pyrene C–H, d, 1H, *J* = 3.6 Hz), 8.17 (pyrene C–H, d, *J* = 3.6 Hz), 8.14 (pyrene C–H, s, 1H), 8.12 (pyrene C–H, s, 1H), 8.05 (pyrene C–H, m, 2H), 8.01 (pyrene C–H, t, 1H, *J* = 7.2 Hz), 7.91 (pyrene C–H, d, 1H, *J* = 7.6 Hz), 3.52 (pyrrolidine C–H, t, 2H, *J* = 6.4 Hz), 3.46 (–CH_2_-CO, t, 2H, *J* = 7.2 Hz), 3.29 (pyrrolidine C–H, t, 2H, *J* = 6.4 Hz), 2.39 (pyrene –CH_2_–, t, 2H, *J* = 7.2 Hz), 2.27 (–CH_2_–CH_2_–CH_2_–, m, 2H), 1.87 (pyrrolidine C–H, m, 4H). ^13^C NMR (100 MHz, CDCl_3_), *δ* (ppm): 171.5, 131.7, 131.2, 130.1, 129.1, 127.7, 127.6, 127.5, 126.8, 126.0, 125.3, 125.2, 125.0, 125.0, 124.9, 123.9, 46.7, 45.9, 34.2, 33.1, 26.9, 26.3, 24.7. FT-IR (cm^−1^): 2925, 1640, 1430, UV-Vis (toluene *λ*_max⁡_): 281, 315, 328, 345. Fluorescence (toluene, *λ*_exc_ = 340 nm, *λ*_em_): 378, 396, 417.

### 4.3. Synthesis of 4-(pyrene-1-yl)-1-([60]fulleropyrrolidine-1-yl)butan-1-one (**2**)

Glycine (11.0 mg, 0.15 mmol), paraformaldehyde (7.0 mg, 0.23 mmol) and C_60_ (39.3 mg, 0.055 mmol) were dissolved in 40 mL dried toluene and stirred for 30 min under N_2_ atmosphere. The solution, still under N_2_ atmosphere, was heated to reflux for 4 hours and then cooled to 0°C. Pyridine (0.12 mL, 1.2 mmol) was added to the solution dropwise followed by 4-(pyrene-1-yl)butanoyl chloride (18.3 mg, 0.06 mmol) in DCM (3 mL). The reaction was stirred for 17 hours at room temperature and the solvent was removed in vacuo. The residue was purified by column chromatography (toluene to toluene/ethyl acetate = 20 : 1), and the crude product was a brownish oil. The product was dissolved in chloroform and precipitated by acetonitrile. The solid was collected and washed with water 3 times to give 2 as a brown solid (4.6 mg, 8% yield over 2 steps). m.p. > 300°C. ^1^H NMR (400 MHz, CDCl_3_), *δ* (ppm): 8.46 (pyrene C–H, d, 1H, *J* = 9.2 Hz), 8.16–8.14 (pyrene C–H, m, 4H), 8.04–7.97 (pyrene C–H, m, 4H), 5.45 (pyrrolidine C–H, s, 2H), 4.99 (pyrrolidine C–H, s, 2H), 3.66 (–CH_2_-CO, t, 2H, *J* = 7.2 Hz), 2.90 (pyrene –CH_2_–, t, 2H, *J* = 7.2 Hz), 2.57 (–CH_2_–CH_2_–CH_2_–, m, 2H). FT-IR (cm^−1^): 2921, 2348, 1651, 1416, 1210, 1182. UV-Vis (toluene, *λ*_max⁡_, nm): 282, 315, 330, 344, 431. Fluorescence (toluene, *λ*_exc_ = 350 nm, *λ*_em_, nm): 391,449.

### 4.4. Graphene Suspensions

Graphene with a distribution of thicknesses (1–10 layers, mean 3–5 layers) was prepared by a modified in-house developed procedure [11]. In a typical preparation, graphite foil (44.5 mg, in approx. 1 × 1 mm pieces) was sonicated in toluene for 10 min using a bench-top bath (VMR Ultrasonic Cleaner, USC500T, HF 45 KHz, 100 W). The remaining graphite foil was removed (43.4 mg after drying in air) and the graphene suspension was allowed to settle for 3 h before using the upper 50% for further experiments or for characterization.

### 4.5. MWCNT Suspensions

Suspensions of MWCNTs were prepared by sonicating purified MWCNTP (1.1 mg) in toluene (5 mL) for 2 min, after which time the suspension was allowed to settle for 1 h before using the upper 25%.

### 4.6. Fluorescence Titration Monitoring the Interaction between Graphene/MWCNT and **1**

From a stock solution of **1** (0.3 mM in toluene), 400 *μ*L was diluted to 20 mL. In a typical functionalization experiment, 1.5 mL of this solution of **1** was added to a 4 mL vial. Then portions (*n* × 100 *μ*L) of graphene suspension (or MWCNT) were added into the vial and, after capping, the mixture was shaken vigorously for a minute (stirring was avoided as that may lead to aggregation) [35]. The suspension was allowed to settle overnight and the upper layer was used for fluorescence spectroscopy. In the control series, pure solvent was added instead.

### 4.7. Sample Preparation for ^1^H NMR Spectroscopy

After registering the ^1^H NMR spectrum of a solution of **1** in CDCl_3_, graphene powder (prepared from the upper layer of a toluene suspension with slow removal of solvent) was added and the mixture was sonicated for 2 min in the benchtop bath. The resulting mixture was examined by ^1^H NMR when the mixture was freshly prepared and after 1 day.

### 4.8. Sample Preparation for Transmission Electron Microscopy (TEM)

Graphene suspensions were treated with pure toluene (sample **a**), [60]fullerene in toluene (sample **b**), or **2** in toluene (sample **c**). The mixtures were shaken (stirring was avoided as that may lead to aggregation) [35] and allowed to settle as described of previously. The solids from the upper layer were collected on a PP filer membrane and washed with pure toluene in order to remove excess [60]fullerene or **2** prior to resuspension in pure toluene by means of a brief bath sonication. The graphene materials were collected on a copper grid with holey carbon support films by means of dip-deposition, a process that samples only what self-assembles on the grid. The grids were handled in air prior to TEM analysis (see Supplementary Materials available online at http://dx.doi.org/10.1155/2013/656185).

## Supplementary Material

Spectra (Uv-vis, IR, NMR) of compounds 1 and 2, titration data and TEM images.
