# Supplementary material for: Noncovalent Functionalization of Graphene in Suspension
Source: ISRN Org Chem. 2013 Mar 28;2013:656185. doi: 10.1155/2013/656185 (PMC3767447; doi:10.1155/2013/656185)
Supplement: Supplementary file 1 — Spectra (Uv-vis, IR, NMR) of compounds 1 and 2, titration data and TEM images. [file 656185.f1.doc]

**Supporting Information**

Non-covalent Functionalization of Graphene in Suspension

Wenzhi Yang, Sultan Akthar,P ^P^Klaus Leifer and H. Grennberg*

**Figure S1**^. 1^H NMR spectrum of compound **1** in CDCl_3_.

**Figure S2** Expansion of the aromatic region of the ^1^H NMR spectrum of compound **1**

**Figure S3** ^1^H NMR spectrum of compound **2** in CDCl_3_

**Figure S4** Expansion of the aromatic region of the ^1^H NMR of compound **2**

**Figure S5** UV-vis spectrum of compound **1** in toluene

**Figure S6** UV-vis spectrum of compound **2** in toluene

**Figure S7** Fluorescence spectrum of compound **1** in toluene

**Figure S8** Fluorescence spectrum of compound **2** in toluene

**Figure S9.** FT-IR spectrum of compound **1**

**Figure S10.** FT-IR spectrum of compound **2**

**Figure S11.** Fluorescence titrations, toluene data for graphene and MWCNT suspensions.

**Figure S12.** HR- TEM of graphene sheets

**Figure S1**P^. 1P^H NMR spectrum of compound **1** in CDClB_3_.

**Figure S2** Expansion of the aromatic region of the P^1P^H NMR spectrum of compound **1**

**Figure S3** P^1P^H NMR spectrum of compound **2** in CDClB_3B_

**Figure S4** Expansion of the aromatic region of the P^1P^H NMR of compound **2**

**Figure S5** UV-vis spectrum of compound **1** in toluene

**Figure S6** UV-vis spectrum of compound **2** in toluene

**Figure S7** Fluorescence spectrum of compound **1** in toluene

**Figure S8** Fluorescence spectrum of compound **2** in toluene

**Figure S9.** FT-IR spectrum of compound **1**

**Figure S10.** FT-IR spectrum of compound **2**

**Figure S11.** Fluorescence titrations, toluene data λB_excB_=344nm upper left: control adding pure solvent, upper right adding graphene suspension, lower left adding MWCNT suspension, lower right fluorescence intensity at 378, 398 and 418 nm. [**1**]≈6.26×10P^-6P^ mol/L, [graphene]≈0.2 mg/mL, [MWCNT]≈0.2 mg/mL,


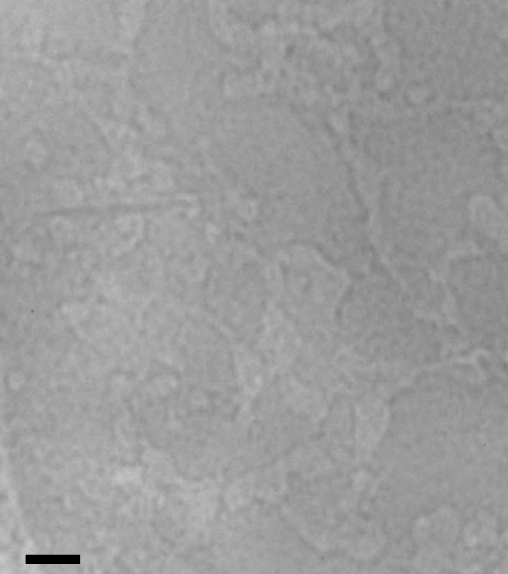

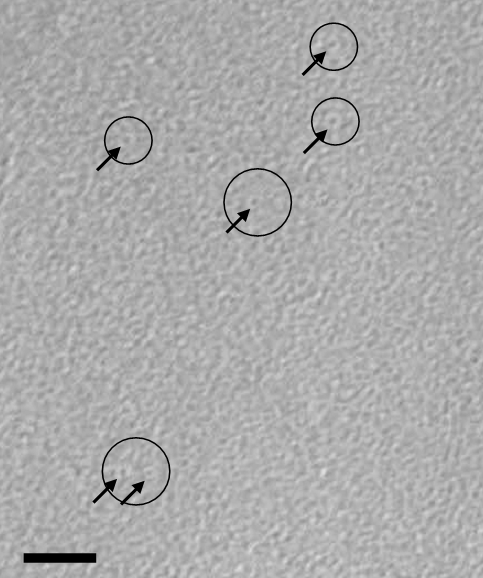


**Figure S12.** Left: HR- TEM of graphene sheets of sample **a** (treated only with solvent). Right: HR-TEM of sample **c**, graphene treated with **2**. Some of the [60]fullerene candidates are encircled. The scale bar corresponds to 5nm in both images.
